# How bio-inspired is your design? A transparent reporting framework

**DOI:** 10.1038/s44172-026-00641-4

**Published:** 2026-04-13

**Authors:** Christina Harvey

**Affiliations:** Department of Mechanical and Aerospace Engineering, University of California, Davis, CA USA

**Keywords:** Engineering, Scientific community, Zoology

## Abstract

From aircraft to neural networks, engineers are often inspired by biological systems; however, literature often conflates inspiration with scientific evidence. Christina Harvey proposes a transparent and consistent reporting framework for bio-inspired design that can accelerate technological progress and stimulate collaborative scientific pursuits.

## Teaser

From aircraft to neural networks, engineers are often inspired by biological systems; however, literature often conflates inspiration with scientific evidence. Here, I propose a transparent and consistent reporting framework for bio-inspired design that can accelerate technological progress and stimulate collaborative scientific pursuits.

Inspiration is a nebulous concept. Being inspired simply requires that an external factor causes some sort of creative mental stimulation. Animals, plants, or other biological systems regularly inspire us with impressive feats that outperform our engineered capabilities. Yet, when we design novel systems to achieve these bio-inspired tasks, there often remains a gap between engineering implementation and biological evidence. For example, many flapping wing mechanisms use constant actuation strategies for wing extension and retraction. However, studies on pigeons revealed that they vary their wing muscle activation patterns in flight^1^, shifting muscle function between that of an actuator, brake, spring or strut^2^. Is this muscular multifunctionality necessary to achieve desirable features of avian-like flapping flight in small-scale aircraft? Maybe, but maybe not. Answering such questions requires clear communication throughout the bio-inspired pipeline, from fundamental research through hypothesis-driven engineering to informed prototype development.

Here, I propose a reporting framework for bio-inspired design that facilitates effective communication by conveying three key elements:

1. the specific source of inspiration,
2. the level of design mimicry, and
3. the strength of biological evidence.

This framework ensures researchers and the public are aware of the provenance and fidelity of the biological information that is embedded into a product or technology without gatekeeping a necessary level of inspiration. For consistency, I largely reference examples from my own field of avian flight and aerial vehicle design but, importantly, this framework is domain agnostic.

## Clarifying terminology: bio-inspired, biomimetics, and bio-informed

To achieve the goal of clear and accurate reporting of bio-inspired design, we must first establish common terminology. Vocabulary is challenging in multidisciplinary fields and, even more so, when existing terminology has historically undergone notable scope creep. In some fields, bio-inspiration, biomimicry, and even bionics are currently treated as synonyms, whereas in others, these terms carry different expectations of biological fidelity.

Generally, bio-inspired design is a “creative approach based on the observation of biological systems”, defined by ISO 18458:2015^3^, an International Standard developed by the International Organization of Standardization Technical Committee in Biomimetics (ISO/TC 266). The standard clarifies that this relation need only be loosely connected to the biological system^3^.

In many disciplines, biomimetics is a narrower category than bio-inspiration. Biomimetic design requires that the designer has, at least attempted to mimic a specific behavior, shape, or morphology, rather than just gain general inspiration from a biological system.

Under the above definitions, neither bio-inspired nor biomimetic design requires a scientific understanding of a biological system’s dynamics, life history, or environmental interactions. For example, an engineer may be inspired after observing a raptor flying between two trees. They could reasonably posit that the bird minimizes energy when flying between the branches, as energy expenditure is often predictive of animal locomotion. They could then impose this expectation as a bio-inspired design requirement. The resulting design may be successful (with sufficient funding and cleverness), although the engineer-imposed requirement may have failed to recognize critical nuances about raptor flight dynamics. For example, there is evidence that hawks minimize their time in a stalled state, rather than their energy expenditure, while approaching a perch^4^. As such, the output design would be the result of an inspired engineer’s intuition and would not be guaranteed to apply the actual functional principles used by the biological system.

Biological insights are valuable, not only because they expand our scientific knowledge, but because they reveal underlying biological principles that can be harnessed to advance technology. To capture this duality, the term bio-informed design has been introduced^5–7^. Bio-informed designs are based on scientific evidence of a fundamental biological principle that is abstracted and then embedded into the design process. This form of design necessitates the use of scientific evidence, which, in turn, requires a hypothesis-driven research method.

## Bio-informed design: embedding the scientific method into the engineering design process

Many leading researchers have developed guidelines for how to implement hypothesis-driven approaches into bio-inspired studies^8–10^. Effective implementation requires an understanding of the scientific method, including an appreciation of the distinction between research questions, hypotheses, and predictions. Research questions capture the broad research goal, such as the many questions that naturally arise while observing biological systems. The scientific method requires that these questions are adapted into hypotheses that: 1) are grounded in literature or existing knowledge, 2) specify an expectation, such as a theorized relationship, pattern, or mechanism, that yields observable predictions, and 3) are testable and falsifiable, ideally achieved by comparing competing hypotheses (including the null). Of note, Full and Koditschek introduced the widely used “templates and anchors” approach^8^, which pairs simplified, physics-informed models (i.e., templates) with higher fidelity realizations of the biological system (i.e., anchors) to test competing hypotheses; for example, alternative spring-mass models enable exploration of bipedal walking principles.

Using such processes, evidence can be obtained to support or reject hypotheses about underlying mechanisms or principles used by biological systems. Once we have scientific evidence of a principle, we can then use engineering tools, such as numerical optimization or rapid prototyping, to explore the consequences of the principle within a context that is directly relevant to the technological application.

While hypothesis-driven studies are the gold standard for fundamental science and necessary for bio-informed approaches, they are not mandatory if a project’s goal is to develop an engineered product. Indeed, the traditional engineering design process can often sidestep the scientific method as it relies on historical data, experiential-based intuition, iterative calculations, and testing.

Yet, engineers benefit from involving scientists throughout a bio-informed process as they help guide the transition from initial inspiration to actionable information^7^. This collaborative environment also allows a synergistic and symbiotic relationship to develop between science and engineering, as engineered systems can provide testbeds to advance biological knowledge^11–13^. For example, robotic systems allow researchers to test whether certain biological traits are sufficient to achieve some desired capability, such as a maneuver, while minimizing natural biological variations that complicate these studies on the true biological systems. These integrative approaches leverage the fields’ relationship to close the loop between engineered design and biological discovery.

## The problem with mistaking inspiration for information

If the traditional engineering design process eventually results in correct or good-enough solutions, what is the harm in conflating biological inspiration with information? Bio-inspired designs based on minimal scientific evidence run a higher risk of being an off-base design point or enforcing wrong, or irrelevant, design constraints. Using uninformed starting conditions and constraints within design processes can produce ineffective designs, waste resources, and slow technological progress.

If our goal is to achieve biological functions that are not possible with current engineered systems, then a bio-informed approach is powerful as biological systems provide an inherent proof-of-concept and a functional starting point within the design process. Successfully embedding scientifically supported, biological principles into the engineering design process may accelerate the innovation cycle and reach a successful design faster than when starting from a clean sheet.

Despite the risk, bio-inspired designs based on minimal scientific evidence can still provide useful insights and should be encouraged, but their reporting needs appropriate hedging. These designs should not be represented as the way a biological system solves a problem, but rather as one proposed way that requires further validation. Interestingly, a recent study found a significant decrease in hedging language in scientific articles over the past two decades, suggesting the shift was associated with academic pressure to produce novel and exciting outcomes^14^. When this trend is combined with the broad public interest in bio-inspired design, this can lead to something we might call bio-washing, akin to greenwashing, wherein a project has higher perceived novelty or impact by including references to bio-inspiration.

## Transparent labelling of bio-inspired design

Tension arises because all bio-inspired studies lie somewhere along a continuum between applied designs based on naïve inspiration from biology and fundamental scientific studies of a biological system. Although these methods are not exclusive, it is easy to accidentally confuse or mislead the public, members in different fields, and new entrants to the field. When bio-inspired design literature is unclear about the provenance and fidelity of their biological claims, it can undermine decades of basic science research and undervalue the field’s potential to advance technology.

**Fig. 1:**
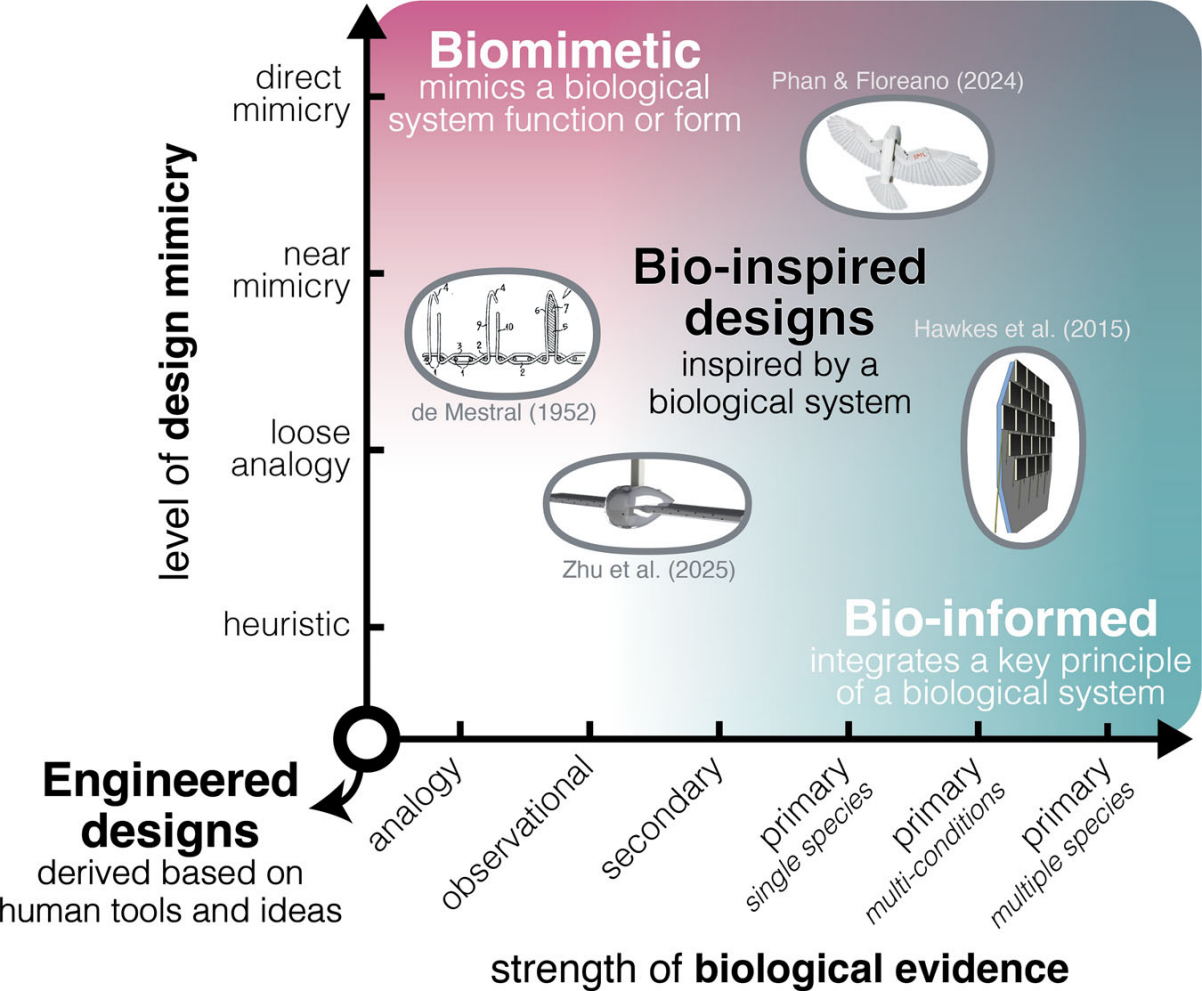
Visualization of suggested minimum reporting requirements for bio-inspired engineering. Examples include Phan and Floreano’s near/direct biomimetic and single species bio-informed drone based on hawk studies^16^, Zhu et al.’s loose analogy and observational/literature-informed wind tunnel model based on the avian nares^15^, de Mestral’s Velcro patent based on analogy/observations with an analogous/near mimicry implementation^17^, and the development of human climbing pads that were informed by many biological studies on geckos, but intentionally reduced the biomimetic complexity to allow scaling of the desired functionality^18,19^. Note the overlapping color ranges in the upper right-hand corner represent designs that are both biomimetic and bio-informed. Phan & Floreano’s image reprinted with permission from AAAS^16^; Zhu et al.’s image reprinted with permission from the authors^15^; Hawkes et al.’s image reprinted with permission from The Royal Society^18^.

**Fig. 2:**
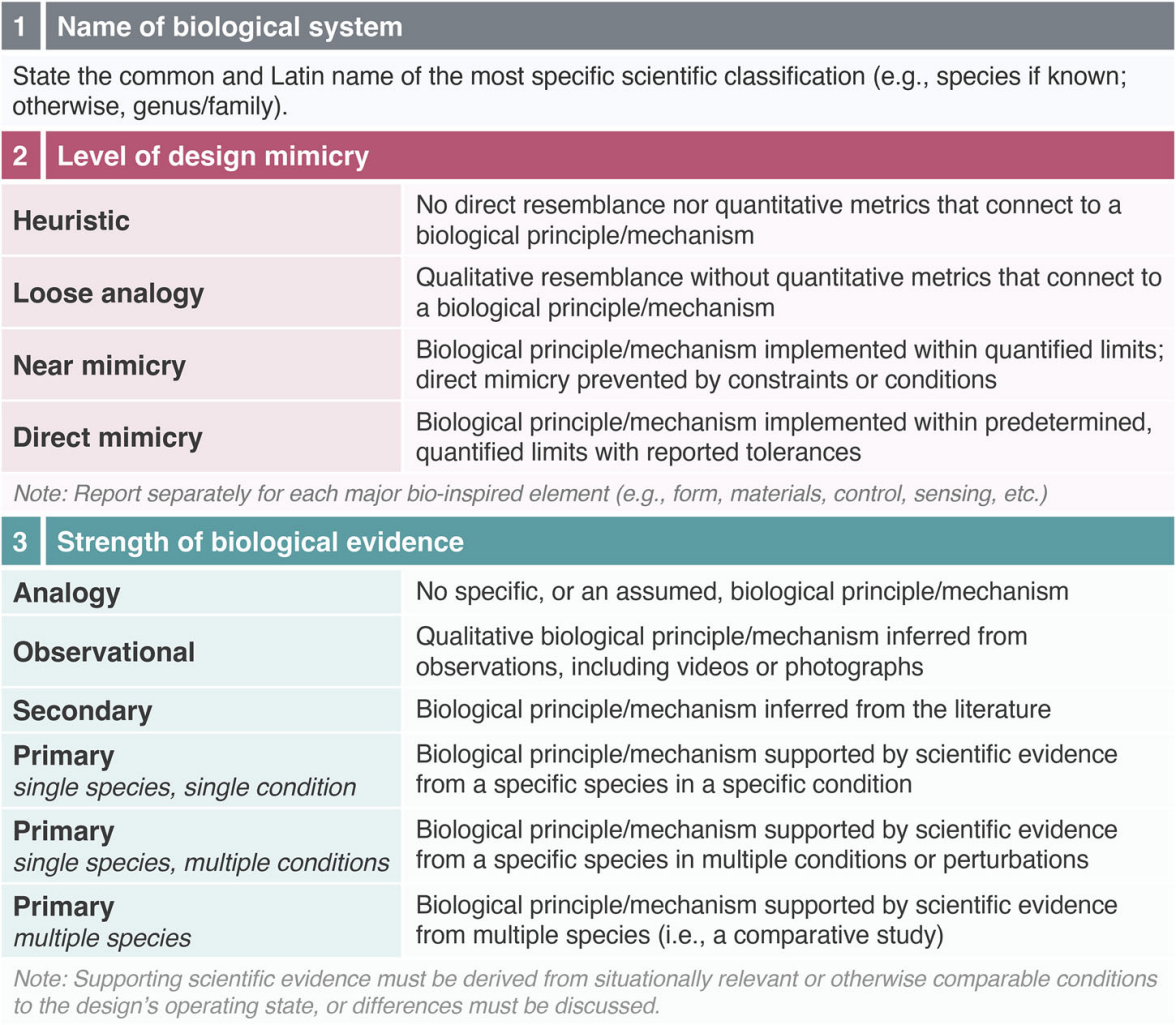
Guidelines of proposed bio-inspired design reporting framework.

As a result, there is a tendency for the peer review process to gate-keep what qualifies as bio-inspired. However, it is pedantic and inefficient to debate whether authors were inspired and how much inspiration is necessary for it to count. Therefore, in line with the ISO standard^3^, I propose that we accept bio-inspiration as a broad, unrestricted term for any inspiration gained from a biological system, but that we require design studies that claim this terminology to also report three key pieces of information: 1) the specific source of inspiration, 2) the level of mimicry in the design, and 3) the strength of biological evidence, as visualized in Fig. 1 and detailed in Fig. 2.

Within this framework, all categories described in Figs. 1, 2 would be labelled as bio-inspired. However, designs would only be labelled as biomimetic when they achieve at least “near mimicry” and as bio-informed when they are based on at least single species-based “primary” biological evidence. Biomimetic and bio-informed are not exclusive terms: if a design successfully incorporates both criteria, it would be placed in the top right-hand corner of Fig. 1. As an example of the framework, I refer to a study from my group^15^ that we deem bio-inspired, but not biomimetic nor bio-informed (Fig. 1):

“Inspired by pelicans (*Pelecanus*), we designed a leader-follower wind tunnel experiment. Our inclusion of nares on the follower model was based on observations and literature inferences that these orifices may function like pressure taps and was achieved by placing taps approximately at the naris’s locations.”

Although this is an inclusive framework, I recommend that authors strongly consider whether it is appropriate to label their work as bio-inspired if the work is a heuristic-only design with analogy-only evidence (i.e., when approaching the lower left-hand corner of Fig. 1).

## Summary

Bio-inspired design has the potential to accelerate technological progress, but this requires transparent and consistent reporting of the design’s source of biological inspiration, level of mimicry, and strength of biological evidence. By adopting this expectation across all levels of the peer review process, we can advance bio-inspired innovation while reducing the risk of artificially constrained solution spaces and weak starting designs. With more transparent bio-inspired literature, incoming researchers can leverage known biological principles to enhance engineering design as well as develop engineering tools that probe our understanding of the natural world.
